# Basophil activation test compared to skin prick test and fluorescence enzyme immunoassay for aeroallergen-specific Immunoglobulin-E

**DOI:** 10.1186/1710-1492-8-1

**Published:** 2012-01-20

**Authors:** Faisal M Khan, Aito Ueno-Yamanouchi, Bazir Serushago, Tom Bowen, Andrew W Lyon, Cathy Lu, Jan Storek

**Affiliations:** 1 Department of Pathology and Laboratory Medicine, University of Calgary, Calgary, Alberta, Canada; 2 Department of Paediatrics, University of Calgary, Calgary, Alberta, Canada; 3 Department of Medicine, University of Calgary, Calgary, Alberta, Canada; 4 Calgary Laboratory Services, Calgary, Alberta, Canada

**Keywords:** Allergic disease, Basophil activation test, Fluorescence enzyme immunoassay, Skin prick test, Aeroallergen, Immunoglobulin-E

## Abstract

**Background:**

Skin prick test (SPT) and fluorescence enzyme immunoassay (FEIA) are widely used for the diagnosis of Immunoglobulin-E (IgE)-mediated allergic disease. Basophil activation test (BAT) could obviate disadvantages of SPT and FEIA. However, it is not known whether BAT gives similar results as SPT or FEIA for aeroallergens.

**Objectives:**

In this study, we compared the results of SPT, BAT and FEIA for different aeroallergens.

**Methods:**

We performed BAT, SPT and FEIA in 41 atopic subjects (symptomatic and with positive SPT for at least 1 of 9 common aeroallergens) and 31 non-atopic subjects (asymptomatic and with negative SPT).

**Results:**

Correlations between SPT and BAT, SPT and FEIA, and BAT and FEIA results were statistically significant but imperfect. Using SPT as the "gold standard", BAT and FEIA were similar in sensitivity. However, BAT had lower specificity than FEIA. False positive (BAT^pos^SPT^neg^) results were frequent in those atopic subjects who were allergic by SPT to a different allergen and rare in non-atopic subjects. The false positivity in atopic subjects was due in part to high levels of serum Total-IgE (T-IgE) levels in atopic individuals that lead to basophil activation upon staining with fluorochrome-labeled anti-IgE.

**Conclusion:**

As an alternative to SPT in persons allergic to aeroallergens, BAT in its present form is useful for distinguishing atopic from non-atopic persons. However, BAT in its present form is less specific than FEIA when determining the allergen which a patient is allergic to. This is due to IgE staining-induced activation of atopic person's basophils and/or nonspecific hyperreactivity of atopic person's basophils.

## Background

Allergen-specific IgE-mediated inflammation is thought to play a major role in the pathogenesis of allergic diseases including extrinsic asthma, rhinitis or eczema. Fc receptors of IgE bind to basophils and mast cells. Crosslinking of the bound IgE by allergens induces the release of inflammatory mediators. Skin prick testing (SPT) is an indirect measure of specific IgE bound to skin mast cells [1]. It has been widely used for the diagnosis of allergic disease because the results are available quickly. However, the utility of SPT is limited in patients with rash or dermographism or those taking antihistamines [2,3]. An alternative to SPT is the determination of serum concentration of specific IgE using the radioallergosorbent test (RAST) or the fluorescent enzymoimmunoassay (FEIA) [4]. A theoretical disadvantage of RAST or FEIA is that it measures both functional IgE (capable of binding to Fcε receptor I [FcεRI] on mast cells or basophils and activating them) and nonfunctional IgE. Another disadvantage of RAST or FEIA is that results are not available as quickly as with SPT.

Basophil activation test (BAT), if shown to be clinically useful, could be automated to the point of giving results within one hour. Moreover, it could theoretically give a more relevant result than RAST or FEIA as only functional IgE (capable of binding to and stimulating basophils) is measured. In the older version of the test, histamine or sulphidoleukotriene release from basophils upon surface-bound IgE crosslinking was measured, which correlated with bronchial provocation test results in asthmatics [5]. In the newer, flow cytometry-based version, a molecule whose expression is up-regulated on basophil surface upon activation (eg, CD63 or CD203c) is detected as a surrogate of inflammatory mediator release [4,6-9]. This is less laborious and faster than sulphidoleukotriene release. On the other hand, Basophil histamine release assay is also faster and automated but can be used only if automated fluorimetry is available. BAT could in some settings be more relevant for the diagnosis of allergic disease than SPT, as allergic inflammation might be mediated by basophils independently of mast cells [10]. Indeed, BAT has been reported to be more sensitive or specific than SPT or RAST/FEIA for allergy to drugs, latex and venoms [11-15]. Here we compared BAT with SPT and FEIA for inhalant allergens.

## Methods

### Subjects

The study was approved by the Ethics Committee of the University of Calgary. Atopic subjects were recruited by allergists (B.S. or T.B.) among patients newly referred to their allergy clinics. Of 45 subjects with symptoms of asthma, rhinitis or eczema recruited, 41 were SPT^pos ^to at least 1 of 9 aeroallergens tested and studied as "atopic subjects". Their median age was 26 years (range, 18-69 years). Asymptomatic subjects (without symptoms of asthma, rhinitis or eczema) were recruited by advertising. Of 42 asymptomatic subjects recruited, 31 were SPT^neg ^for all 9 aeroallergens tested and studied as "non-atopic subjects". Their median age was 29 years (range, 15-47 years). To ensure uniformity in assessing the presence of symptoms of asthma, rhinitis or eczema between the symptomatic and asymptomatic persons, the International Study of Asthma and allergies in Childhood questionnaire (version Phase II, http://isaac.auckland.ac.nz/PhaseOne/Manual/ManFrame.html, accessed December 27, 2007) was used for both the symptomatic and asymptomatic subjects. A written informed consent was obtained from all the subjects. Presence of symptoms was defined as a positive answer to question # 2 (Have you had wheezing or whistling in the chest in the last 12 months?), #7 (In the last 12 months, has your chest sounded wheezy during or after exercise?) or #8 (In the last 12 months, have you had a dry cough at night, apart from a cough associated with a cold or chest infection?) of the asthma section; question # 2 (In the past 12 months, have you had a problem with sneezing, or a runny, or blocked nose when you did not have a cold or the flu?) of the rhinitis section or question # 2 (Have you had this itchy rash at any time in the last 12 months?) of the eczema section of the questionnaire. None of the subjects had cancer, autoimmune disease or immunodeficiency, and none had ever received allergen immunotherapy, systemic immunosuppressive drugs in the past 3 months or antihistamines in the 7 days prior to SPT. None of the patients were treated with omalizumab before blood sample collection. Blood was drawn for BAT and FEIA prior to SPT (typically within one hour prior to SPT) to eliminate the possibility of SPT influence on the result of BAT or FEIA.

### Allergens

Allergen extracts (ALK-Abello, Horsholm, Denmark, except for Timothy grass pollen extract from Greer Laboratories, Lenoir, NC, USA) were kindly donated by Western Allergy, Mississauga, Ontario, Canada. Neat extracts contained 50% glycerol and 0.4% phenol. Negative control was 0.9% sodium chloride in 50% glycerol and 0.4% phenol (Glycerol Saline). Positive control was histamine 1 mg/mL in 50% glycerol and 0.4% phenol (Histatrol, [ALK-Abello, Horsholm, Denmark]) for skin prick test and anti-FcεRI (Alpco Diagnostics, Salem, NH, USA) for BAT. The allergen concentration used for SPT was in compliance with the US guidelines on probable effective concentration range for allergen extracts (http://www.aaaai.org/professionals/resources/immunotherapy/, accessed on November 26, 2009). The allergen concentration used for BAT was based on our preliminary experiments in which BAT was performed for each allergen using five different concentrations - 5-times, 10-times, 50-times, 100-times and 1000-times lower concentration than that used for SPT. The 100-times lower concentration was associated with the highest percentage of stimulated (CD63+) basophils above Glycerol Saline background. The final concentration of all 9 allergens used for SPT and BAT is mentioned in Table 1:

**Table 1:** Concentration of allergens used for SPT and BAT

Allergen	Concentration of the allergen	
	**SPT**	**BAT**

Cat pelt	10,000 BAU/ml	100 BAU/ml

Dog epithelium	1:20	1:2000

*Dermatophagoides pteronyssius (DP*)	1000 AU/ml	100 AU/ml

*Dermatophagoides farinae (DF)*	1000 AU/ml	100 AU/ml

Alternaria	1:10	1:1000

Hormodendrum/Cladosporium	1:10	1:1000

Timothy grass pollen	100,000 AU/ml	1000 AU/ml [BAT]

Short ragweed pollen	1:20	1:2000

Birch tree (*Betula verrucosa*) pollen	1:20	1:2000

### Skin Prick Testing

Allergens and positive and negative control drops were applied on the volar forearms with at least 2 cm distance from each other. For each allergen, a single epicutaneous prick was performed using Allersharp^® ^device (Western Allergy, Mississauga, Ontario, Canada). Wheal area was recorded for Histatrol at 10 min, and for others (each allergen and negative control) at 15 min by outlining the area with a felt-tipped pen, and transferring the outline onto 3 M tapes to keep a permanent record of SPT. The recorded wheal areas were scanned as jpeg files and analyzed by Image J software (National Institutes of Health, Bethesda, MD, USA) to determine the average diameter of each wheal. The average diameter of the negative control wheal was subtracted from each allergen wheal (corrected diameter). The SPT result was considered positive if the corrected diameter was greater than 3 mm compared to a negative saline control [16]. All subjects had a valid SPT as defined by at least 1 mm diameter difference between the positive and negative controls [16].

### Basophil Activation Test

**Figure 1:**
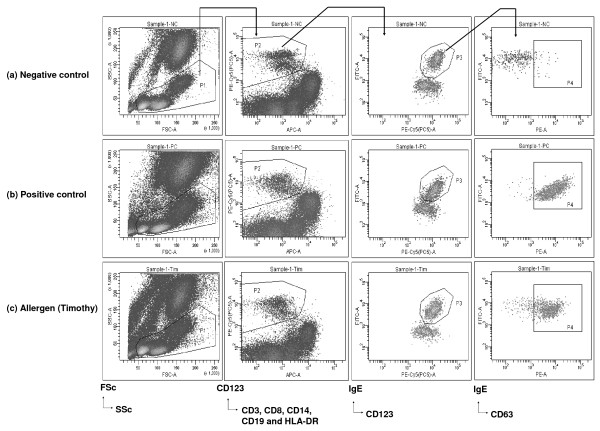
**Example of basophil activation assay**. Buffy coat cells suspended in an interleukin-3 (IL-3)-containing buffer were stimulated with (a) negative control (Glycerol Saline); (b) positive control (anti-FcεRI) and (c) Timothy allergen. Basophills were defined as cells not expressing CD3, CD8, CD14, CD19 or HLA-DR (P2) and expressing CD123 and IgE (P3). Activated basophils were defined as CD63+ basophils (P4).

Twenty mL of heparinized peripheral blood were centrifuged to obtain buffy coat cells, which were suspended in interleukin-3 (IL-3)-containing buffer (Alpco Diagnostics, Salem, NH, USA). The suspension (100 uL) was incubated with allergen (see "Allergens", above, for concentration) or negative control (Glycerol Saline) or positive control (anti-FcεRI) for 25 min at 37°C in humidified atmosphere. Then the cells were stained for 30 min at 4°C with the following fluorochrome-labeled antibodies: IgE-FITC (Serotec, Raleigh, NC, USA), CD63-PE (Beckman Coulter, Mississauga, Ontario, Canada), CD123-PECy5 (BD Biosciences, San Jose, CA, USA), and CD3-APC, CD8-APC, CD14-APC, CD19-APC, and HLA-DR-APC (Beckman Coulter, Mississauga, Ontario, Canada). Red blood cells were lysed using ammonium chloride lysis buffer. After washing and resuspending the cells in PBS with 1% bovine serum albumin and 0.1% sodium azide, the cells were analyzed by flow cytometry (FACS Aria, BD Biosciences, San Jose, CA, USA). The number of events acquired was set to contain at least 200 basophils (expressing IgE and CD123 and not expressing CD3, CD8, CD14, CD19 or HLA-DR). Percent activated (CD63+) basophils were expressed for each allergen. This percentage was corrected for nonspecific activation by subtracting the percent activated (CD63+) basophils in the negative control (Figure 1). If the corrected percentage was at least 15% for cat and timothy, 25% for DP, and 30% for dog and birch, BAT for that allergen was considered positive (for rationale, see results).

**Figure 2:**
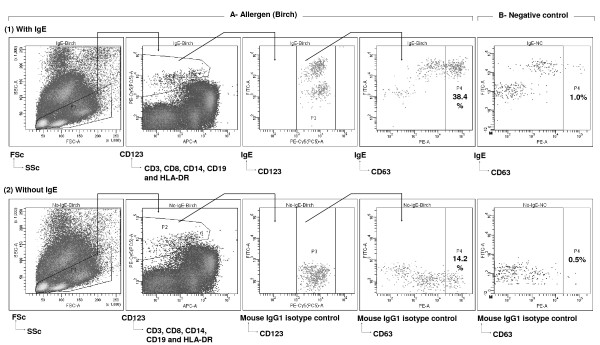
**Basophil activation assay performed (1) with or (2) without IgE staining**. Buffy coat cells suspended in an IL-3-containing buffer were (A) stimulated with birch allergen or (B) not stimulated (negative control - Glycerol Saline). Subsequently, the cells were stained with (1) IgE-FITC, CD63-PE, CD123-PC5, and HLA-DR/CD3/CD19-APC or (2) IgG1 Mouse Isotype Control-FITC, CD63-PE, CD123-PC5, and HLA-DR/CD3/CD19-APC. Basophills were defined as cells that do not express CD3, CD8, CD14, CD19 or HLA-DR (P2), but express CD123 (P3). Activated basophils were defined as CD63+ basophils (P4). Density plot displaying only P4 is shown for the negative control for both versions of staining (with or without IgE). Percentages of activated basophils are shown in the respective P4 gates. Corrected percentage of activated basophils was calculated by subtracting saline control percentage from allergen stimulated percentage of activated (CD63+) basophils. In this example, the corrected percentage of activated basophils on stimulation with Birch is 37.4% (38.4-1.0%) when anti-IgE was used for basophil staining and is 13.7% (14.2-0.5%) when anti-IgE was not used for basophil staining.

To evaluate whether staining of basophils for cell surface-bound IgE with fluorochrome-labeled anti-IgE induces CD63 upregulation, we performed BAT both with and without fluorochrome-labeled anti-IgE (Figure 2). In the version without the anti-IgE, basophils were identified as cells expressing CD123 and not expressing CD3, CD8, CD14, CD19 or HLA-DR. The percentages of activated (CD63+) basophils yielded by the two versions of staining were compared in 14 subjects (8 atopic and 6 non-atopic).

### Fluorescent enzymoimmunoassay

Sera from the research subjects were stored in tightly sealed vials at -86°C. Near the end of the study, allergen-specific IgE concentration was determined using UniCAP100 instrument and specific IgE FEIA reagents (Phadia, Uppsala, Sweden, accessed January 7, 2008) per manufacturer instructions. For economy, IgE for only the most common allergens was tested: Cat epithelium and dander (e1), Dog epithelium (e2), *D. pteronyssinus *(d1), *D. farinae *(d2), Timothy grass pollen (g6), and Birch tree (*Betula verrucosa*) pollen (t3). Allergen-specific IgE FEIA values of 0.1 kU/L or higher were considered positive.

### Measurement of Total-IgE

Total-IgE (T-IgE) was measured in the sera of 36 atopic and 28 non-atopic individuals on the Roche Elecsys 2010 analyzer by electro-chemiluminescence immunoassay using the manufacturer's recommended operating procedures, reagents and calibrations (Roche Diagnostics Canada, Laval, QC).

### Statistics

Correlations were analyzed using Spearman's rank order correlation coefficient test. Significance of difference of test results between 2 subject groups was tested using Mann-Whitney-Wilcoxon rank sum test. The choice of a non-parametric test to compare two groups (Mann-Whitney-Wilcoxon rank sum test) was based on the fact that data distribution in at least one of the group in all but one comparison was non-Gaussian. In order to maintain the consistency, Mann-Whitney-Wilcoxon rank sum test was used for all comparisons. In the experiments comparing the effect of IgE staining on CD63 upregulation, Wilcoxon matched paired test was used to test significance of difference between the subject groups stained with or without IgE.

Two-tailed P values less than 0.05 were considered significant.

## Results

Of the 72 subjects (41 atopic and 31 non-atopic subjects), 67 (93%) were evaluable for BAT (39 atopic and 28 non-atopic). The remaining 5 subjects (2 atopic and 3 non-atopic) were not evaluable because of unresponsive basophils (less than 15% basophils expressing CD63 above background upon stimulation with anti-FcεRI [positive control]). The subjects with unresponsive basophils were eliminated from data analysis. Of the 39 evaluable atopic subjects, 11 (28%) had asthma, rhinitis and eczema, 13 (33%) had asthma and rhinitis, 3 (8%) had rhinitis and eczema, 9 (23%) had rhinitis only, 2 (5%) had asthma only, and 1 (3%) had eczema only. By SPT, 19 (49%) of the 39 evaluable atopic subjects were allergic to cat, 14 (36%) to dog, 11 (28%) to *Dermatophagoides(D) pteronyssinus*, 8 (20%) to *Derpmatophagoides(D) farinae*, 2 (5%) to Alternaria, 2 (5%) to Hormodendrum, 26 (67%) to Timothy grass, 21 (54%) to birch tree and 10 (26%) to short ragweed. Median number of positive skin prick tests per atopic subject was 2. Given the small number of subjects atopic to *D.farinae*, Alternaria, Hormodendrum and short ragweed, only analyses pertinent to cat, dog, *D.pteronyssimus*, Timothy grass and birch tree are presented here.

### Correlation between BAT and SPT and between FEIA and SPT

Correlations between BAT and SPT results for all 5 allergens tested were statistically significant but imperfect, as correlation coefficients ranged from 0.37 to 0.54 (Table 1). Correlations between FEIA and SPT results were also statistically significant; the correlation coefficients were consistently higher than for BAT and SPT, ranging from 0.42 to 0.77 (Table 2).

**Table 2:** Correlation between SPT and BAT, SPT and FEIA, and BAT and FEIA results, and sensitivity* and specificity** of BAT and FEIA using SPT as the "gold standard".

	SPT vs BAT	SPT vs FEIA	BAT vs FEIA	BAT sensitivity	FEIA sensitivity	BAT specificity	FEIA specificity
Cat	R = .42 P < .001	R = .61 P < .001	R = .44 P < .001	.84	.84	.73	.83

Dog	R = .37 P = .001	R = .59 P < .001	R = .32 P = .007	.57	.64	.81	.92

DP	R = .38 P = .001	R = .42 P < .001	R = .33 P = .005	.63	.63	.76	.87

Timothy	R = .54 P < .001	R = .77 P < .001	R = .53 P < .001	.80	.80	.80	.82

Birch	R = .43 P < .001	R = .52 P < .001	R = .35 P = .003	.57	.61	.76	.93

* Calculated as the number of persons allergic for that allergen by both SPT and BAT or FEIA/total number of persons allergic for that allergen by SPT.

** Calculated as the number of persons nonallergic for that allergen by both SPT and BAT or FEIA/total number of persons nonallergic for that allergen by SPT. Persons nonallergic for that allergen by SPT included both asymptomatic persons with negative SPT for all 9 allergens as well as symptomatic persons with positive SPT for allergen(s) other than the allergen of interest.

### Comparison of specificities of BAT and FEIA

**Figure 3:**
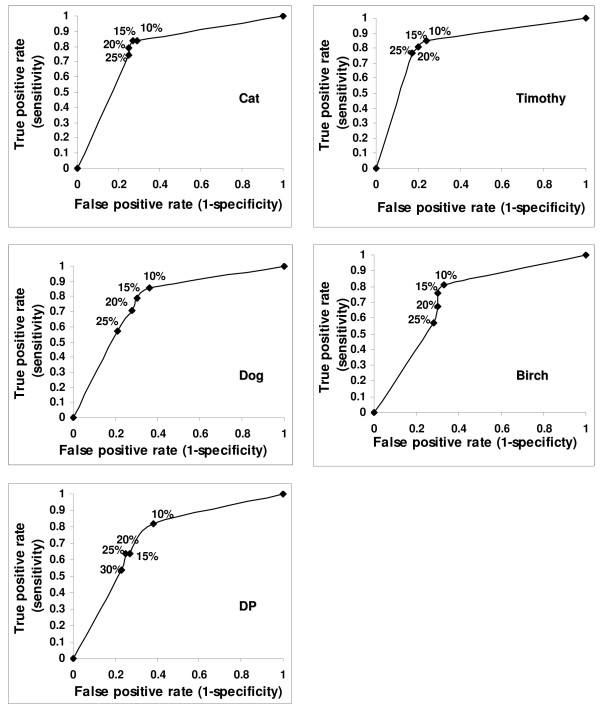
**Receiver operating characteristics (ROC) curves used for the determining the cutoffs for positive BAT results**. Sensitivity and Specificity of BAT was calculated using different cutoffs (10%, 15%, 20%, 25% and 30% above background) for positivity. The cutoffs at which sensitivity of BAT was similar to FEIA were selected. Selected cutoff value included 15% above background for Cat and Timothy, 25% for DP and 30% for Dog and Birch.

To formally compare the specificity of BAT and FEIA (using SPT as the "gold standard"), we first set the cutoffs for BAT positivity for each allergen in such a way that the sensitivities of BAT and FEIA were similar. We used BAT receiver operating characteristic (ROC) curves for choosing the cutoffs (Figure 3). Thus, the sensitivities of BAT and FEIA were intentionally similar (Table 1). Then we calculated the specificities, using the same cutoffs. For all 5 allergens, the specificity of BAT was lower than the specificity of FEIA (Table 2) due to a relatively high number of BAT^pos^SPT^neg ^results.

Further, we reanalyzed the data with allergen-specific IgE FEIA values of 0.35 kU/L or higher being considered positive. Accordingly, the cutoffs for BAT positivity were selected by plotting ROC curves to keep the sensitivities of BAT and FEIA were similar. However, consistent with earlier results, the specificity of BAT for all 5 allergens was found lower than the specificity of FEIA (Additional File 1).

### BAT^pos^SPT^neg ^("false positive") results were frequent in atopic but infrequent in non-atopic individuals

**Figure 4:**
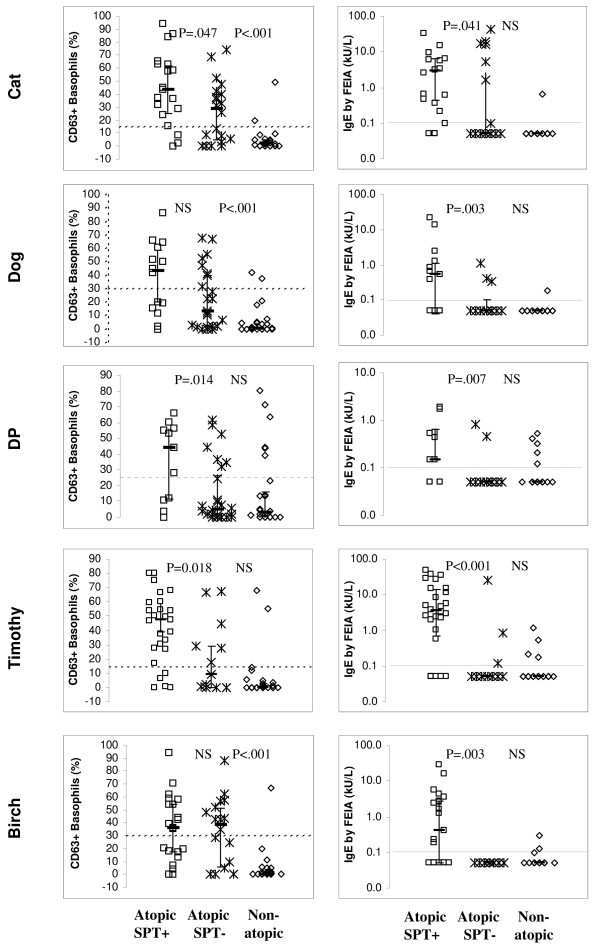
**Results of BAT *(left) *and FEIA *(right) *in atopic patients (n = 39, squares and asterisks) and non-atopic persons (n = 28, diamonds)**. The atopic patients are divided into those allergic to the allergen of interest per SPT result ("Atopic SPT^pos^", squares) and those allergic to a different allergen(s) ("Atopic SPT^neg^", asterisks). The numbers of Atopic SPT+ patients were 19 for cat, 14 for dog, 11 for *D.pteronyssimus*, 26 for Timothy, and 21 for birch. The numbers of Atopic SPT^neg ^patients can be calculated for each allergen as 39 minus the number of Atopic SPT^pos ^patients (eg, 39-19 = 20 for cat). Significance of the difference between the Atopic SPT^pos ^and Atopic SPT^neg ^groups and between Atopic SPT^neg ^and Non-atopic groups is given in the upper section of each plot. BAT results are displayed as corrected percentage of activated (CD63+) basophils (saline control percentage subtracted). Undetectable IgE levels by FEIA are displayed as 0.05 kU/L. Dashed lines denote cutoffs for positivity. Cutoff for FEIA positivity was 0.1 kU/L, while that for BAT varied for different allergens - 15% above background for Cat and Timothy, 25% for DP and 30% for Dog and Birch.

The BAT^pos^SPT^neg ^results were frequent in atopic subjects who were SPT^neg ^for the allergen of interest (but SPT^pos ^for another allergen) and rare in non-atopic subjects (Figure 4, *left*). Also, median percentages of activated (CD63+) basophils were higher in the atopic SPT^neg ^subjects than the non-atopic subjects (the difference was significant for cat, dog and birch - Figure 4, *left*), whereas median IgE levels by FEIA were similar in the atopic SPT^neg ^patients and the non-atopic persons (Figure 4, *right*). Thus, the basophils of atopic individuals were more "excitable" (prone to upregulate CD63) than basophils of non-atopic individuals.

Consistent with the nonspecific excitability of atopic patients' basophils, Figure 4 also shows that whereas the difference in allergen-specific IgE was high between SPT^pos ^and SPT^neg ^atopic patients (and significant for all 5 allergens), the difference in percent stimulated basophils was relatively low between SPT^pos ^and SPT^neg ^atopic patients (and significant for only 3/5 allergens). This could be due to the fact that SPT^pos ^patients were defined as those having symptoms of asthma/rhinitis/eczema (irrespective of exposure to an allergen) and positive skin prick test to an allergen, but not necessarily symptoms upon exposure to that allergen. Thus, we re-analyzed the analyses presented in Figure 4, except defined the SPT^pos ^patients more strictly, ie, as patients with symptoms of asthma/rhinitis/eczema AND positive SPT for the allergen AND clinical history consistent with allergy to that allergen (at a minimum, perennial symptoms for a perennial allergen or seasonal symptoms for a seasonal allergen). Even with this stricter definition of SPT^pos ^atopic patients, the difference in percent stimulated basophils was relatively low between SPT^pos ^and SPT^neg ^atopic patients (and significant for only 2/5 allergens) (Additional File 2).

### Effect of anti-IgE staining on BAT results

**Figure 5:**
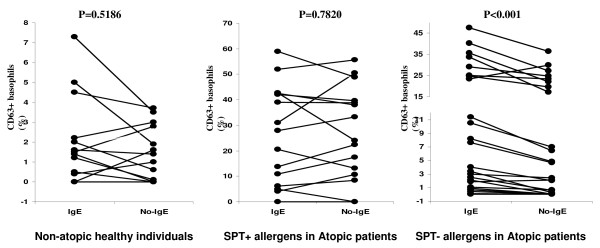
**Comparison of BAT performed with vs without IgE in healthy (non-atopic) persons and atopic patients**. The atopic patients are divided into those atopic to the allergen of interest per SPT result (middle) and those allergic to a different allergen(s) (right). Significance of the difference between groups stained with and without IgE is given in the upper section of each plot. BAT results are displayed as corrected percentage of activated (CD63+) basophils (saline control percentage subtracted).

A potential reason for the basophils of atopic individuals being more "excitable" could be the following: The staining of basophils with fluorochrome-labeled anti-IgE could partially activate basophils by crosslinking IgE (specific for any antigen) bound to basophils [17]. This is generally thought to be negligible as the staining is done at 4°C. However, if this is not negligible, basophils of atopic individuals may be activated by the anti-IgE staining to a significant degree whereas basophils of non-atopic individuals to a nonsignificant degree. This is because atopic individuals have on average ~10-fold higher serum levels of total IgE (T-IgE) than non-atopic individuals [18,19] and thus more IgE is expected to be bound to basophils of atopic than non-atopic individuals. To evaluate whether the staining with anti-IgE induces significant upregulation of CD63 on the basophils of atopic individuals, we compared BAT results performed with and without staining with fluorochrome-labeled anti-IgE. Upregulation of CD63 on the basophils of the atopic patients in case of those allergens for which patients were not allergic to, was significantly higher when cells were stained with fluorochrome-labeled anti-IgE in comparison to the scenario when cells were not stained with anti-IgE (P < 0.001, Figure 5, right). This suggests that the anti-IgE staining induces CD63 upregulation even at 4°C if a relatively large amount of total IgE is bound to basophils. On the contrary, the BAT results were similar with and without anti-IgE staining in the non-atopic individuals (Figure 5, left); thus, CD63 may not be significantly upregulated by the anti-IgE staining if only a small amount of total IgE is bound to basophils. The results were similar with and without anti-IgE staining also in the atopic patients in case of allergens the patients were allergic to (Figure 5, middle), suggesting that the stimulation through allergen-induced crosslinking of specific IgE at 37°C is dominant (not significantly enhanced by the anti-IgE antibody-induced crosslinking of total IgE at 4°C).

### Effect of total IgE levels on BAT results

**Figure 6:**
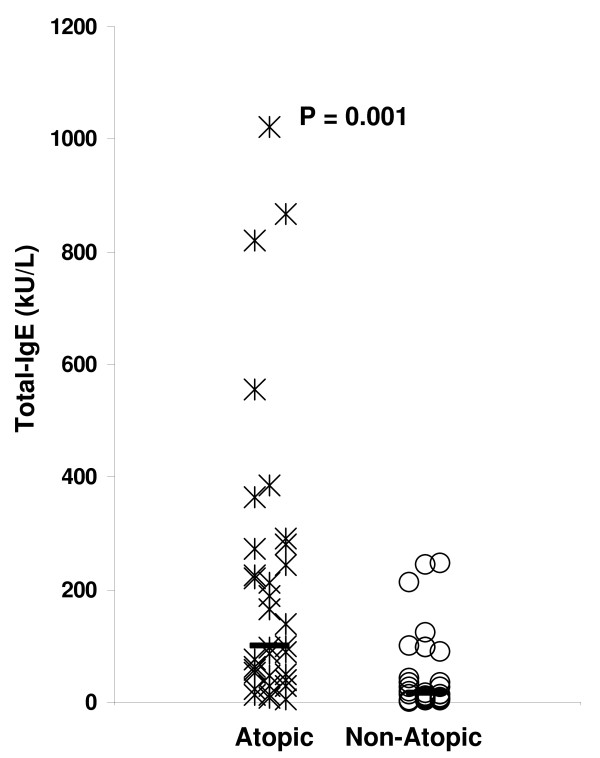
**Comparison of T-IgE levels in atopic patients and non-atopic individuals**. T-IgE levels were measured in 36 atopic patients and 28 non-atopic individuals. Significance of the difference between the two groups is given in the upper section of the plot.

**Figure 7:**
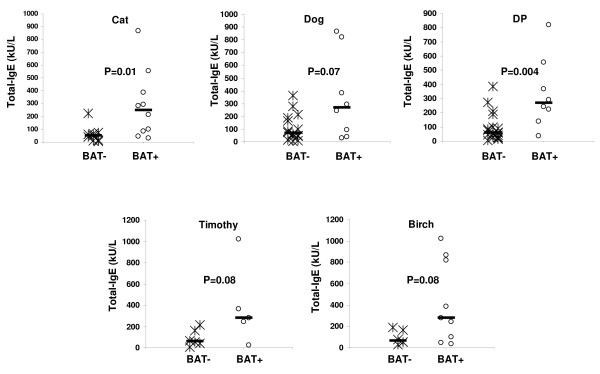
**Comparison of T-IgE levels in **BAT^pos^SPT^neg ^**and **BAT^neg^SPT^neg ^**atopic patients**. The numbers of SPT^neg ^atopic patients analyzed for T-IgE levels were 19 for cat, 24 for dog, 27 for *D.pteronyssimus*, 11 for Timothy, and 16 for birch. Significance of the difference between the two groups is given in the upper section of the plot.

As suggested in the above experiment (Figure 5), a potential reason for the nonspecific increase in CD63 upregulation of basophils of SPT^neg ^atopic individuals compared to non-atopic individuals may be a high amount of T-IgE in the SPT^neg ^atopic individuals and thus a high amount of T-IgE bound to the basophils of the SPT^neg ^atopic individuals. During staining of basophils for surface-bound IgE with fluorochrome-labeled anti-IgE, the high amount of T-IgE on basophils could lead to nonspecific CD63 upregulation in the SPT^neg ^atopic individuals. To evaluate this hypothesis, we measured T-IgE levels in both atopic and non-atopic individuals (Figure 6). As expected, T-IgE levels were significantly higher (p = 0.001) in atopic (median, 98.6 kU/L; range, 5.7 -1022.0 kU/L) than non-atopic (median, 16.4 kU/L; range, 0.2-245.8 kU/L) individuals. Among SPT^neg ^atopic individuals allergic to cat, dog, DP, Timothy or birch, T-IgE levels were higher in BAT^pos^SPT^neg ^individuals than BAT^neg^SPT^neg ^individuals (Figure 7). The difference was statistically significant for cat (p = 0.01) and DP (p = 0.004) and marginally significant for dog (p = 0.07), Timothy (p = 0.08) and birch (P = 0.08). In conclusion, the increased reactivity of basophils from SPT^neg ^atopic individuals compared to non-atopic individuals may be related to the high amount of T-IgE bound to the their basophils.

## Discussion

Our results suggest that BAT is as good as SPT or FEIA for distinguishing atopic from non-atopic persons. However, in identifying the allergen to which an atopic person is allergic, BAT in its present form appears to be inferior to SPT or FEIA. When SPT is considered as the "gold standard" and when the cutoffs are set so that BAT and FEIA have similar sensitivities, BAT has lower specificity than FEIA. The BAT^pos^SPT^neg ^(false positive) results are frequent in atopic but rare in non-atopic individuals. This suggests that basophils from atopic subjects are hyperresponsive to a nonspecific stimulus. The results did not changed when we included the additional 5 subjects (2 atopic and 3 non-atopic) which were not included in the primary analysis as they have unresponsive basophils (less than 15% basophils expressing CD63 above background upon stimulation with anti- FcεRI (data not shown).

The nonspecific stimulus does not appear to come from the IL-3-containing buffer, phenol or glycerol, as the negative control BAT results were not higher in the atopic subjects than in non-atopic subjects (median 10.1% vs 4.8% CD63+ basophils, P = 0.44) and because BAT results were found similar when only saline was compared with phenol and glycerol saline as a negative control in three individuals (median 6.1% vs 7.8% CD63+ basophils, P = 0.82). Consistent with that, in a previous report, IL-3 did not alter CD63 expression on basophils incubated with IL-3 in the absence of allergen; IL-3 only potentiated CD63 upregulation induced by allergen [20]. Could the high occurrence of BAT^pos^SPT^neg ^results in atopic patients be due to an intrinsic hyperresponsiveness of atopic persons' basophils? Positive control (anti- FcεRI) BAT results were not significantly higher in the atopic subjects compared to non-atopic subjects (median 59.2% vs 54.8% CD63+ basophils above background, P = 0.66). This suggests that either atopic person's basophils are not intrinsically hyperresponsive or that if they are intrinsically more "excitable" than non-atopic persons' basophils, this is not apparent when a strong stimulus like anti- FcεRI is used.

The nonspecific stimulus may be the staining of basophils for surface-bound IgE with fluorochrome-labeled anti-IgE. Atopic patients have higher serum levels of total IgE than non-atopic individuals (Figure 6) [18,19]. The pair wise comparison of CD63 expression between two versions of staining (with and without anti-IgE) consistently showed a higher percent of activated (CD63+) basophils (Figure 4, right) in the atopic SPT^neg ^patients when staining with anti-IgE. Thus, fluorochrome-labeled anti-IgE can stimulate basophils coated with IgE even if staining is done at 4^°^C. This appears to be significant only in atopic patients and not non-atopic individuals, possibly due to the presumed higher IgE serum levels and thus higher density of basophil surface-bound IgE in atopic compared to non-atopic individuals. Use of a fixative like paraformaldehyde after the stimulation and before staining, staining in a calcium-free buffer, a blood wash before staining or use of other basophil marker like CD203c may be helpful in overcoming the non-specific stimulation due to IgE staining [21-24].

The finding of significantly higher levels of T-IgE in BAT^pos^SPT^neg ^than BAT^neg^SPT^neg ^atopic individuals (Figure 7) further supports the contention that the basophils of SPT^neg ^atopic individuals are overreactive due to the high amount of basophil-bound IgE crosslinked during staining with fluorochrome-labeled anti-IgE. However, the hyperreactivity of atopic persons' basophils may not be due only to the activation by the fluorochrome-conjugated anti-IgE, as 4 of 8 atopic patients tested showed a high percentage (> 15%) of activated basophils for SPT^neg ^allergen(s) even when we used staining without IgE. It should be determined whether BAT could better discriminate the allergen to which a patient is allergic to by using basophils from non-atopic individuals (mixed with patient serum).

A limitation of this study is the fact that BAT and FEIA were compared to SPT, which is not a definitive diagnostic test, as 29-46% asymptomatic persons are SPT^pos ^for at least one of 10 common allergens,[25] and some patients with provocation tests or clinical history strongly suggesting allergic disease due to a specific allergen and elevated serum levels of IgE to that allergen are SPT^neg ^for that allergen [26,27]. Thus, it is conceivable that at least some of the BAT^pos^SPT^neg ^results represent false negative SPT rather than false positive BAT results. Future studies could include bronchial or nasal provocation tests as the "gold standard". An alternative would be to include intracutaneous testing in BAT^pos^SPT^neg ^patients. Allergen specific IgE may be more specific than SPT [28], though defining a cut off point for specific IgE levels to diagnose allergic disease has been challenging [29]. As clinical history may represent a better way of determining the clinical relevance of an allergen [30,31], we compared the results of BAT and FEIA for atopic individuals defined by clinical history in addition of being SPT^pos ^and having symptoms of symptoms of rhinitis/asthma/eczema. The results of this additional analysis (Additional File 2) were consistent with BAT in its present form being less specific than FEIA when determining the allergen to which a patient is allergic.

It is also important to note that although negative control BAT results were similar in atopic and non-atopic subjects but this observation alone cannot completely rule out the role of IL-3 as a potent inducer of CD63 upregulation. It is still possible that the addition of IL-3 could also increase the sensitivity to allergens that are seemingly negative in SPT or basophils from atopic subjects might be responding directly to IL-3. Therefore, another limitation of the study is that we have not analyzed all BAT results with and without IL-3 which might have reduced the number of false-positive BAT results.

Based on these results, we conclude that when SPT is used as the "gold standard", both BAT and FEIA could distinguish an atopic from non-atopic persons, but BAT is inferior to FEIA in distinguishing the allergen to which an atopic person is allergic, from other allergens for which the same atopic person is not allergic. This is in part because basophils from at least some atopic individuals appear to be nonspecifically hyperresponsive, due at least in part to IgE bound to basophils. It is important to mention here that we have not measured the levels of Spleen tyrosine kinase (Syk), a key regulatory factor in the IgE-mediated allergic signal transduction pathway in mast cells and basophils [32]. Theoritically, it is possible that hyperresponsiveness of basophills may not be due to the high levels of basophil-bound IgE but due to high level of Syk. Another reason for the inferiority of BAT as a diagnostic tests is the fact that it cannot be generalized to the population since basophils of many individuals are unresponsive [7]. Further improvements of the assay should focus on avoiding the anti-IgE staining induced basophil stimulation (eg, by staining fixed basophils) or focus on basophil histamine release instead of an up-regulation of CD63 as functional marker of basoiphil stimulation.

## Competing interests

The authors declare that they have no competing interests.

## Authors' contributions

FMK and AU-Y performed experiments. AU-Y performed analysis and generated the initial draft of the manuscript, FMK performed experiments, carried out final analyses and wrote later drafts of the manuscript, BS and TB recruited atopic subjects and performed skin prick tests, AL was involved in the experiments and analysis related to FEIA and T-IgE measurement, CL was involved in performing laboratory experiments assay, JS designed the study, edited the drafts and the final version of the manuscript. All authors have read and approved the final manuscript.

## Supplementary Material

Additional file 1 Sensitivity* and specificity** of BAT and FEIA (positive cut off as 0.35 kU/L) using SPT as the "gold standard". Click here for file

Additional file 2 **Results of BAT *(left) *and FEIA *(right) *in atopic patients (n = 34, squares and asterisks) and non-atopic persons (n = 28, diamonds) based on clinical history and SPT results**. The atopic patients are divided into those allergic to the allergen of interest per SPT and clinical history ("Atopic SPT^pos^", squares) and those allergic to a different allergen(s) ("Atopic SPT^neg^", asterisks). The numbers of Atopic SPT+ patients were 15 for cat, 10 for dog, 8 for *D.pteronyssimus*, 22 for Timothy, and 15 for birch. The numbers of Atopic SPT^neg ^patients can be calculated for each allergen as 34 minus the number of Atopic SPT^pos ^patients (eg, 34-15 = 19 for cat). Significance of the difference between the Atopic SPT^pos ^and Atopic SPT^neg ^groups and between Atopic SPT^neg ^and Non-atopic groups is given in the upper section of each plot. BAT results are displayed as corrected percentage of activated (CD63+) basophils (saline control percentage subtracted). Undetectable IgE levels by FEIA are displayed as 0.05 kU/L. Dashed lines denote cutoffs for positivity. Cutoff for FEIA positivity was 0.1 kU/L, while that for BAT varied for different allergens - 15% above background for Cat and Timothy, 25% for DP and 30% for Dog and Birch. Click here for file
