# Supplementary material for: Basophil activation test compared to skin prick test and fluorescence enzyme immunoassay for aeroallergen-specific Immunoglobulin-E
Source: Allergy Asthma Clin Immunol. 2012 Jan 20;8(1):1. doi: 10.1186/1710-1492-8-1 (PMC3398323; doi:10.1186/1710-1492-8-1)
Supplement: Additional file 1 — Sensitivity* and specificity** of BAT and FEIA (positive cut off as 0.35 kU/L) using SPT as the "gold standard". [file 1710-1492-8-1-S1.DOC]

**Additional File 1. S**ensitivity* and specificity** of BAT and FEIA (positive cut off as 0.35 kU/L) using SPT as the “gold standard”.

|  | Sensitivity | | | | Specificity | |
| --- | --- | --- | --- | --- | --- | --- |
|  | BAT | | FEIA | | BAT | FEIA |
| Cat | .74 | .74 | | .75 | | .85 |
| Dog | .62 | .69 | | .79 | | .90 |
| DP | .58 | .45 | | .77 | | .93 |
| Timothy | .80 | .80 | | .80 | | .88 |
| Birch | .57 | .52 | | .76 | | 1.00 |

* Calculated as the number of persons allergic for that allergen by both SPT and BAT or FEIA / total number of persons allergic for that allergen by SPT.

** Calculated as the number of persons nonallergic for that allergen by both SPT and BAT or FEIA / total number of persons nonallergic for that allergen by SPT.

The cutoffs for BAT positivity were selected in such a way that the sensitivities of BAT and FEIA were similar. BAT receiver operating characteristic (ROC) curves were plotted for choosing the cutoffs. Selected cutoff value included 25% above background for cat, dog and birch, 15% for Timothy and 30% for DP.
