# Supplementary figures and images for: Basophil activation test compared to skin prick test and fluorescence enzyme immunoassay for aeroallergen-specific Immunoglobulin-E
Source: Allergy Asthma Clin Immunol. 2012 Jan 20;8(1):1. doi: 10.1186/1710-1492-8-1 (PMC3398323; doi:10.1186/1710-1492-8-1)

Cat

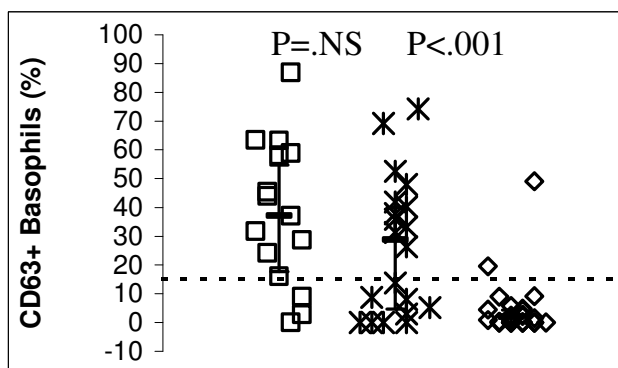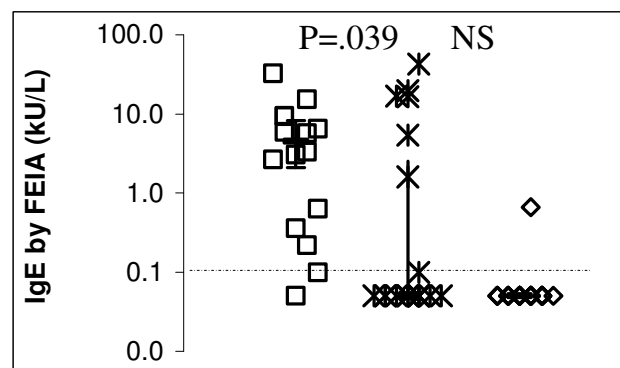

Dog

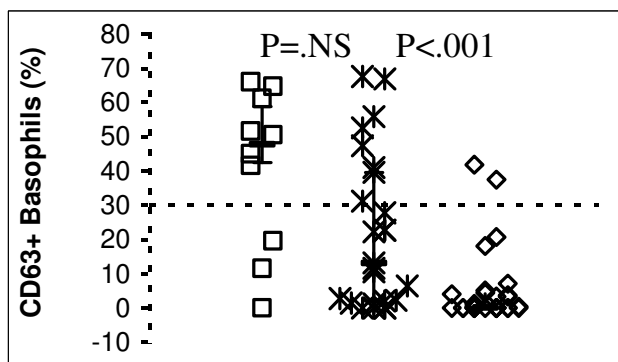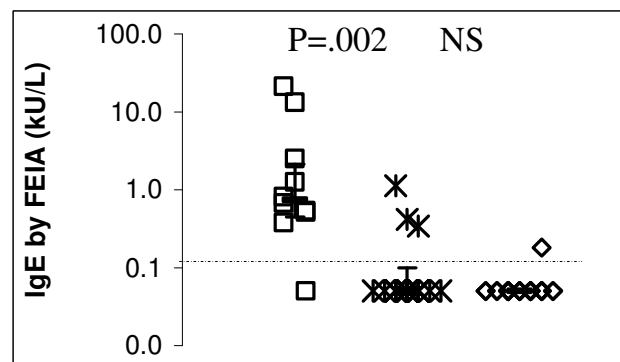

DP

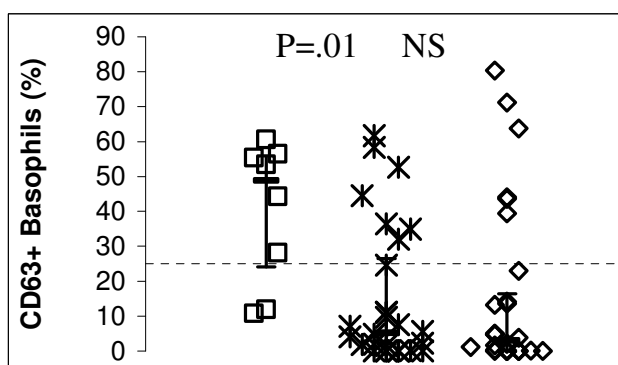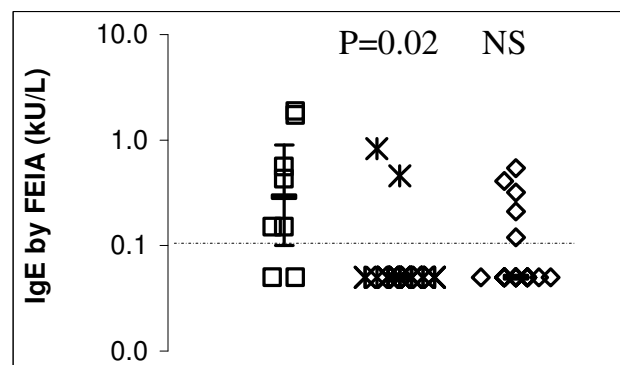

Timothy

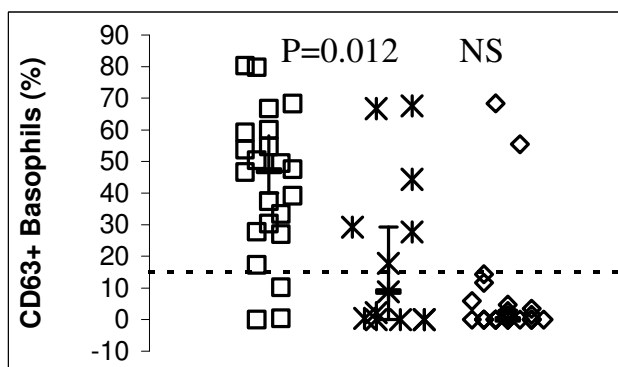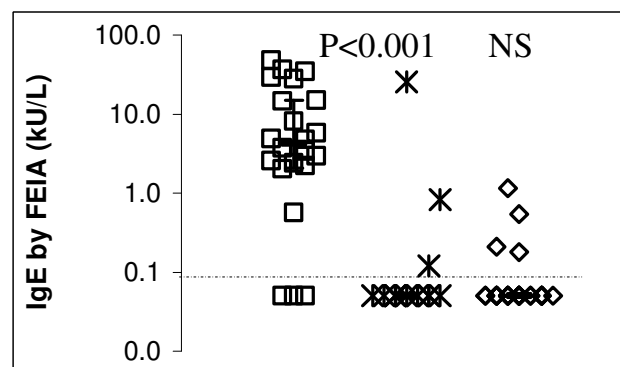

Birch

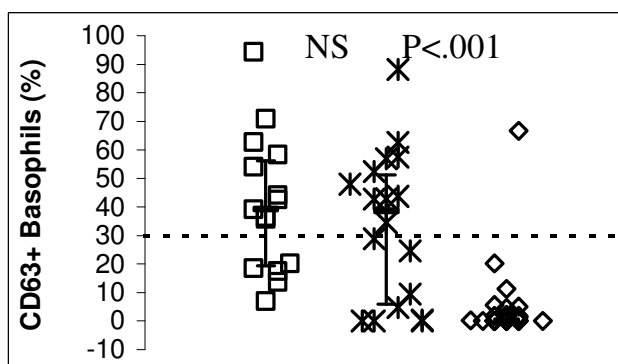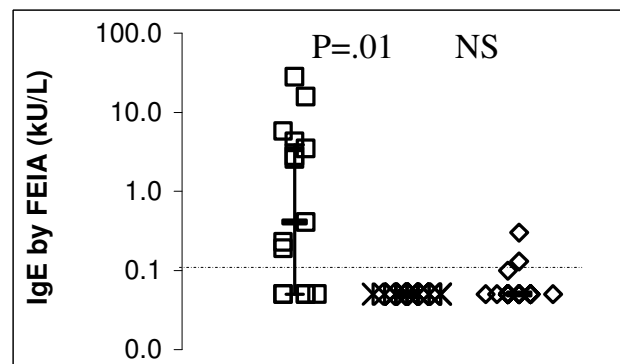

Atopic SPT+ Atopic SPT- Non-Atopic

Atopic SPT+ Atopic SPT- Non-Atopic

Supplement: Additional file 2 — Results of BAT (left) and FEIA (right) in atopic patients (n = 34, squares and asterisks) and non-atopic persons (n = 28, diamonds) based on clinical history and SPT results. The atopic patients are divided into those allergic to the allergen of interest per SPT and clinical history ("Atopic SPTpos", squares) and those allergic to a different allergen(s) ("Atopic SPTneg", asterisks). The numbers of Atopic SPT+ patients were 15 for cat, 10 for dog, 8 for D.pteronyssimus, 22 for Timothy, and 15 for birch. The numbers of Atopic SPTneg patients can be calculated for each allergen as 34 minus the number of Atopic SPTpos patients (eg, 34-15 = 19 for cat). Significance of the difference between the Atopic SPTpos and Atopic SPTneg groups and between Atopic SPTneg and Non-atopic groups is given in the upper section of each plot. BAT results are displayed as corrected percentage of activated (CD63+) basophils (saline control percentage subtracted). Undetectable IgE levels by FEIA are displayed as 0.05 kU/L. Dashed lines denote cutoffs for positivity. Cutoff for FEIA positivity was 0.1 kU/L, while that for BAT varied for different allergens - 15% above background for Cat and Timothy, 25% for DP and 30% for Dog and Birch. [file 1710-1492-8-1-S2.PDF]
